# Evidence for contamination with *C*. *trachomatis* in the household environment of children with active Trachoma: A cross-sectional study in Kongwa, Tanzania

**DOI:** 10.1371/journal.pntd.0007834

**Published:** 2019-12-23

**Authors:** Sheila K. West, Afshan A. Nanji, Harran Mkocha, Beatriz Munoz, Charlotte Gaydos, Thomas C. Quinn

**Affiliations:** 1 Dana Center for Preventive Ophthalmology, Johns Hopkins School of Medicine, Baltimore Maryland, United States of America; 2 Kongwa Trachoma Project, Kongwa, Tanzania; 3 Division of Infectious Diseases, Johns Hopkins School of Medicine, Baltimore, Maryland, United States of America; 4 Division of Intramural Research, National Institute of Allergy and Infectious Diseases, National Institute of Health, Bethesda, Maryland, United States of America; London School of Hygiene and Tropical Medicine, UNITED KINGDOM

## Abstract

**Background:**

Trachoma, a conjunctivitis caused by repeated infections with *Chlamydia trachomatis*, remains a significant cause of blindness worldwide. While mass treatments with azithromycin decreases disease and infection, re-emergence occurs, indicating that elimination may require other sustainable interventions. Environmental changes largely focus on facial hygiene and latrines, but further work to identify other possible transmission targets are needed. We sought to determine, in a cross-sectional survey of households of children with active trachoma, if we could detect the presence of *Chlamydia trachomatis* on household objects and on family members based on sleeping and caretaking patterns.

**Methods:**

In five villages in Kongwa, Tanzania, children <five years were randomly chosen for examination for trachoma, and households of all children with active trachoma (n = 90) were eligible for this study. We administered structured questionnaires of sleeping and caretaking habits. Based on the responses, environmental swabs of bedding, furniture, clothing, and hands were taken and processed using Amplicor for detecting *C*. *trachomatis* DNA.

**Results:**

Of 80 visited households, 13 (16%) had at least one swab from environmental sources positive for *C*. *trachomatis* DNA. A positive environmental swab was associated with the presence of ocular infection in the index child (Odds Ratio = 22.0, p = .007), the presence of an infant <1 year of age in the household, and whether the children’s clothing had not been recently washed.

**Conclusions:**

*C*. *trachomatis* DNA is present in the environment of children with active trachoma, especially in households with an ocular infection. Specific findings also suggest that washing hands, clothing, and bedding may be important.

## Author summary

Trachoma is the leading infectious cause of blindness world-wide. It is a conjunctivitis caused by repeated infections with *Chlamydia trachomatis*, spread from person to person by contact with infected ocular/nasal secretions. While mass treatment with azithromycin decreases infection, re-emergence occurs, indicating that elimination may require other sustainable interventions. Environmental changes largely focus on facial hygiene and latrines, but further work to identify other possible transmission targets is needed. In this cross-sectional study of households where at least one child has trachoma, we found evidence of *C*. *trachomatis* DNA on several environmental sources including clothing, bedsheets, furniture, and hands. Besides the presence of infection with *C*. *trachomatis* in the index child, chlamydial DNA in the environment was also associated with the presence of an infant in the household. Washing clothes within the past three days was associated with not having *C*. *trachomatis* DNA in the environment. Our study provides suggestive evidence that in high prevalence villages, *C*. *trachomatis* may be contaminating environmental objects, including hands and clothing. These findings suggest that behavior change communication around hygiene may need to be enlarged to focus on keeping ocular and nasal secretions off faces and hands, and promoting washing of clothing as well.

## Introduction

Trachoma, a disease caused by repeated infection with ocular serovars of *Chlamydia trachomatis*, is the world’s leading cause of infectious blindness [1]. It is thought to be endemic in 43 countries, mainly in Africa, and almost exclusively affects the most impoverished regions of the world [2]. The pathogenesis of trachoma begins with repeated ocular *C*. *trachomatis* infections, leading to inflammation and follicles on the upper tarsal conjunctiva. Over years of re-infection, these initial changes may proceed to scarring, trichiasis (inward upper lid turning as a result of fibrosis of the tarsal conjunctiva), and ultimately, corneal opacity and blindness.

In response to this large public health problem, the World Health Organization has recommended the SAFE strategy for trachoma elimination. This approach consists of surgery for trichiasis, antibiotics for treatment of infection, facial cleanliness, and environmental interventions to for sustained reduction of transmission [3]. Alone, mass drug administration (MDA) with Azithromycin dramatically decreases rates of infection in communities, but multiple annual rounds of MDA are required in hyperendemic areas, which can be very slow to show reduction in infection and clinical disease [4–6]. Evidence for re-emergence and the slow rate of decline indicates that parallel and sustainable interventions are likely needed in addition to antibiotics for disease control.

*C*. *trachomatis* is present in the ocular and nasal secretions of infected persons, and is the only route for the spread of infection as there is no animal or insect reservoir. Re-emergence of trachoma following mass treatment occurs in spatial clusters, suggesting that household and other close contacts contribute to disease spread [7]. Personal interactions, principally involving pre-school children who are the reservoir of the disease in hyperendemic communities [8], facilitate transmission of *C*. *trachomatis*. Maintaining facial hygiene and environmental improvements, largely focused on reduction of flies through latrine construction, have been promoted to reduce access to infected secretions [9–15]. However, other possible routes of transmission of infected secretions, such as on hands of caretakers or clothing and bedding, may be present and could serve as targets for interventions.

In this study, we identified children with active trachoma and went to their households to find evidence of the presence of *C*. *trachomatis* DNA on household objects, clothing and hands of family members, in order to understand potential environmental sources of disease transmission.

## Methods

### Household and index child identification

The current study was part of a larger two year study of trachoma prevalence performed in several districts in Tanzania that was evaluating the effects of multiple rounds of mass drug administration [16]. In that study, a census was conducted to enumerate all households within each village. Households with children age 0-5yrs were randomly chosen for participation. In households with multiple children in this age range, one child was randomly selected for inclusion. These children served as sentinel children for determination of the prevalence of active trachoma. The current cross-sectional study was conducted in the five villages in Kongwa district that were involved in the main study and surveyed between April 7 –May 7, 2007. These villages were: Banyi Banyi, Majawanga, Makawa, Mkutani, and Njoge. We took advantage of the main study for data on the census in these villages, and the survey that identified the trachoma status of children. All sentinel children found to have active trachoma and their households were eligible for the current study and the children will be referred to henceforth as “index” children. The sample size was based on determining the precision of finding at least one household with a positive environmental sample, set at proportion = .01 with a precision of +/- .01. In this case and without accounting for clustering, N = 96. We used the number of villages that could be visited in one month (n = 5), and expected rate of trachoma (15%) that would determine the number of households visited of all households surveyed (between 80–110). As this was the first study to determine environmental contamination from trachoma, there was no a priori determination of number of samples that needed to be taken from each household.

### Ocular examination and ocular sample analysis

Ocular exams to identify the presence of trachoma were conducted for all sentinel children within a village on a single day. An experienced trachoma grader, with 2.5x loupes, assessed the presence of clinical trachoma using the WHO simplified grading system [17]. In this system, “active trachoma” is defined as either trachomatous inflammation–follicular (TF) or–intense (TI), where TF is the presence of five or more follicles of at least 0.5mm diameter on the upper tarsal conjunctiva and TI is pronounced inflammatory thickening of the tarsal conjunctiva that obscures more than half of the normal deep tarsal vessels.

An ocular swab for detection of *C*. *trachomatis* infection was taken of the index children in the home. For this, a sterile Dacron swab was rubbed across the upper left conjunctiva three times and placed in a sterile, dry vial. A standard protocol was used in the field to avoid contamination. The individual who swabbed the eyelid was the only person to touch the swab at any point and never touched the children or any other individual during the procedure. A separate individual flipped the eyelids of the children, switching gloves between each child. “Air” swabs were taken at random, about 1 per 50 examinations and tested masked to the status as control swabs. The swab-containing vials were frozen and stored in a -20 degree freezer until shipped to the International Chlamydia Laboratory at the Johns Hopkins Hospital in Baltimore, MD. They were tested for the presence of *C*. *trachomatis* DNA using a, qualitative polymerase chain reaction assay, Amplicor (Roche Molecular Systems, Indianapolis, IN) according to the manufacturer’s directions. Samples were considered negative if they had an optical density of <0.2 A_450_ and positive if ≥0.8 A_450_. Samples with optical densities ≥0.2 and <0.8, were considered equivocal and retested; no sample had equivocal results upon retesting. All “air” swab controls were negative, indicating no contamination.

### Questionnaire administration

In order to guide the collection of environmental samples to test for the presence of chlamydia DNA, we developed a questionnaire on the sleeping and caretaking arrangements of all children age five and under in a household. Questions included where each child slept and with whom, who took care of each child during the day, how ocular and nasal secretions were cleaned or each child, and frequency of washing of bedding and clothing (collected as <1 day, <3 days, < one week, one week or more ago). Questions were directed to the individual caretakers. The houses of the index children were visited the day after the survey. All questions were asked by a local Tanzanian interviewer, fluent in the local dialect. Questionnaire information was also supplemented by observation. For example, if any child was observed touching a household object, we added that potential site for swabbing. Observations were only made during three time points: the greetings as the staff came to the house, during the conduct of the questionnaire, and while swabbing sites identified from the questionnaire. Relevance and comprehension of the questions were tested during a pilot study on women with children from a village not included in this study.

We attempted to visit the household of each index child for interviewer administration of the questionnaire. Each household in which the inhabitants were absent was visited at least twice on two separate days in an attempt to collect information.

### Household environment sample collection

Based on the answers obtained from the questionnaire, one person (AN) determined where to target areas of the household (bedding, clothing, hands, etc.) as potential sources of *C*. *trachomatis* DNA for environmental swab collection. These areas were sprayed with sterile water, and a sterile Dacron swab was rubbed against the damp material and then placed into sterile, dry vials. Samples were collected as follows: samples from where the child slept were collected from an area of one foot by one foot, at the location of where the child’s head rested while sleeping; for bedding, the area of collection was approximately one foot by six inches near the head of the bed; furniture (bed or wall poles, stools, etc.) samples were obtained from a one foot by six inch area in which ocular and nasal secretions were said to be wiped; clothing samples were collected from a smaller area of six inches by two inches at the location of the child’s head when carried or where secretions were said to be wiped; those from the hand were collected from the entire palmar surface and in between the fingers. The extent of the area to be swabbed was arbitrary but standardized by use as there are no published data on collecting environmental samples. All vials were frozen and shipped to Johns Hopkins for PCR analysis, as described for the ocular exam vials.

### Data analysis

The primary outcome was presence of *C*. *trachomatis* DNA in the environment, defined as having one or more PCR-positive environment samples from the household. Data collected on potential risk factors for environmental exposure was summed across each household. (e.g. particular child and caretaking behaviors were considered to be performed in the household if any individual living there answered “yes” to the respective question).

Data was analyzed using STATA version 10.0. Frequency of washing clothes was categorized as <3 days ago versus more than 3 days. The last washing of bedding was asked as less than a week, or a week or more ago. Unadjusted analyses of association between household risk factors and the outcome were done using the Fisher’s exact test. A multivariate model was created using logistic regression and backwards elimination employed to remove factors if shown to be statistically insignificant, using a pre-specified criterion p-value of 0.1.

### Ethical approval

Ethical approval for this study was obtained from the Johns Hopkins University Institutional Review Board and the National Institute for Medical Research in Tanzania. Written informed consent for study participation was obtained from the child’s guardian, and oral consent was re-obtained for conducting swabbing of surfaces at the home, as approved by the National Institute for Medical Research. All consents were obtained following the tenets of the Declaration of Helsinki.

## Results

A total of 669 children age ≤5 years in the five villages received an ocular examination. Active trachoma was identified in 90 of these children. Prevalence of trachoma in those ≤5 years of age varied by village (Table 1) from more than 20% to under 10%.

**Table 1:** Trachoma prevalence in villages and visited households.

Village Name	# children examined to identify index cases of trachoma	Number of Children with active trachoma (% of total examined)	# Households Visited	Number of Index Children in visited households who are PCR positive (% infected)
Makawa	124	12 (9.7)	11	5 (45.5)
Banyi Banyi	132	15 (11.4)	13	1 (7.7)
Majawanga	133	16 (12.0)	14	8 (57.1)
Njoge	128	35 (27.3)	33	17 (51.5)
Mkutani	152	12 (7.9)	9	2 (22.2)
Total	669	90 (13.5)	80	33 (41.3)

Of the 90 households in which an index child with active trachoma was present, we were able to collect questionnaire information from 80 (Table 1). In the remaining 10 households, no one was present on at least two separate occasions. In no household was either the questionnaire or sampling of environmental sources refused, and we had no missing data from either source. The prevalence of infection by village in the index children ranged from 8% to 57%, with no swabs missing.

Overall, the group was 47.5% male and 20% were age ≤ 1 years (Table 2). Approximately half of the children had another sibling in the household who was also less than 5 years but in no household was there more than three children between the ages of 0–5 years.

**Table 2:** Characteristics of children examined (n = 80).

Characteristic	N (Percent)
Male	38 (47.5)
Age: ≤1 year 2–3 years 4–5 years	16 (20.0) 43 (53.6) 21 (26.3)
Has Sibling age <5 years	41 (51.3)
Sleeping partner Sleeps alone Sleeps with other child ages 0–5 years* Sleeps with other children ages 6–9 years* Sleeps with parents only	1 (1.3) 31 (39.2) 8 (10.0) 43 (53.8)
Sleeping location Sleeps on a bed Sleeps on an animal skin on the floor Sleeps on a mat on the floor	74 (92.5) 3 (3.8) 3 (3.8)
Bed Covers* Bedsheets/blanket Kanga	62 (77.5) 19 (23.8)
Carried by caretaker Of those ever carried, carried in a kanga* Of those ever carried, carried on the hip* Of those ever carried, carried on the back*	62 (77.5) 53 (85.5) 59 (95.2) 48 (77.4)
Cleaning of eye and nasal secretions* Hands used to clean secretions Clothing used to clean secretions Separate cloth used to clean secretions Water used to clean secretions	79 (98.8) 70 (87.5) 4 (5.0) 2 (2.5)

* not mutually exclusive

Almost all of the index children slept with at least one other person, and 46% of the children slept with at least one other child, predominantly between ages 0–5 years. More than 90% of children slept on an actual bed, rather than on animal skin, mat, or clothes on the floor.

Three-quarters of the children were carried by their caretakers except for older children ages 4–5 years who were not carried. Children were carried in multiple ways, with carriage on the hip being the most common, but followed closely by carriage on the caretaker’s back. Eighty-six percent of children, regardless of where they were carried by the caretaker, were carried using a kanga, a large rectangular cloth.

Ocular and nasal secretions were cleaned by both caretakers and the children themselves in a variety of ways. For all but one index child, one of the methods used involved use of hands, either the child’s or caretaker’s to wipe away secretions. In the majority of cases (88%), either the child or caretaker’s clothing was also used for cleaning. Secretions were rarely wiped using a separate cloth (5%) or washed with water (2.5%).

**Fig 1:**
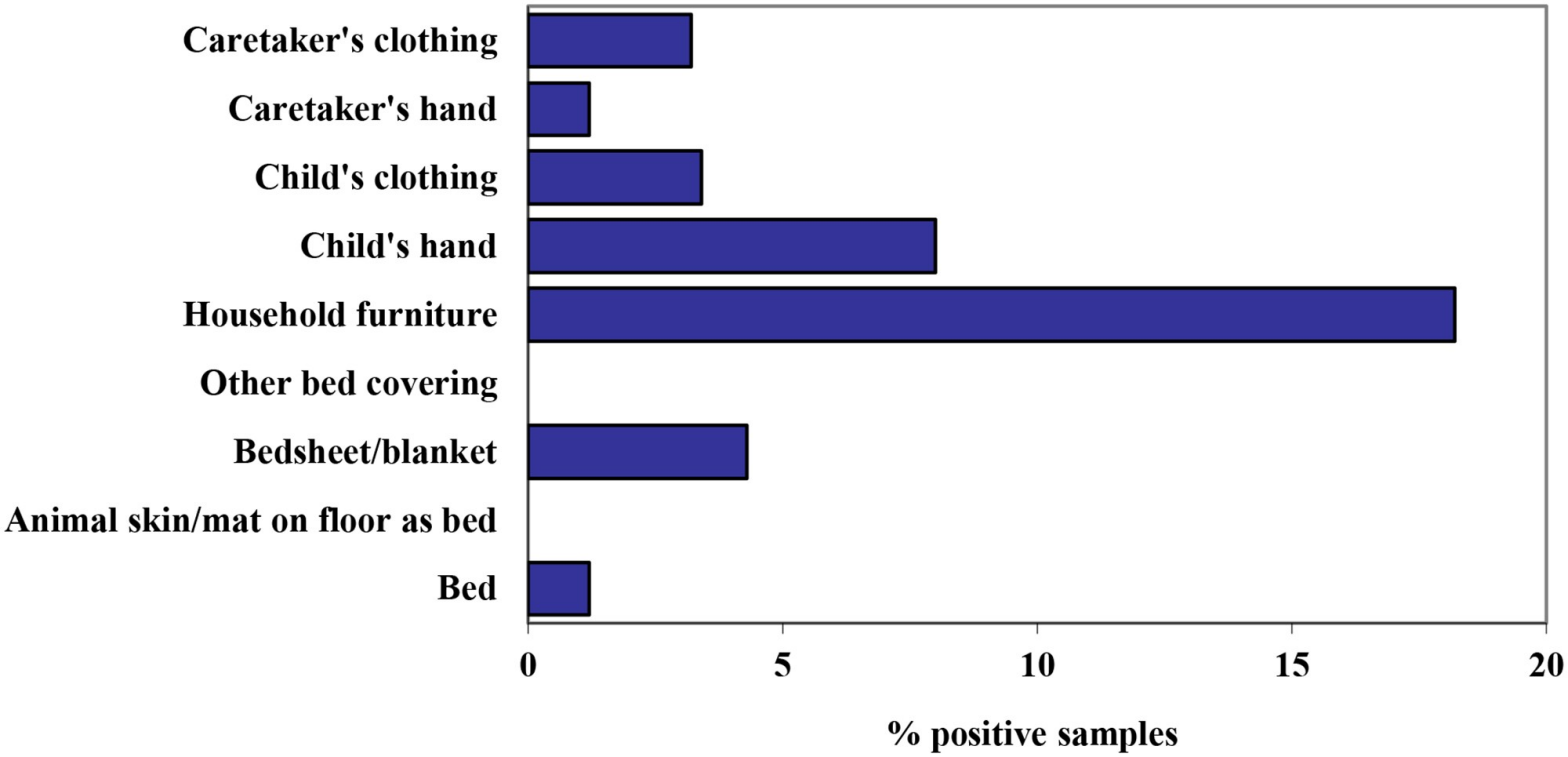
Environmental sources of PCR-positive environmental samples, over all samples taken for that source.

There were 23 positive environmental swabs out of the 633 taken (3.6%). The average number of samples taken per household was 7.9 and every household had at least 4 samples taken. Samples of the household environment showed that *C*. *trachomatis* DNA was present on household objects, children, and adults (Fig 1). Locations with the highest proportion of positive swabs were household furniture (18%) and children’s hands (8%). Other sources of positive samples were bedsheets/blankets (4%), children’s clothing (3%), caretaker’s clothing (3%), beds (1%), and caretaker’s hands (1%).

The 23 positive environmental swabs were obtained from 13 households (Table 3). Twenty-one of the positive swabs, collected from 11 households, were from the same village, Njoge. The villages of Majawanga and Makawa each contributed one positive environmental sample and the remaining two villages yielded none.

**Table 3:** Description of 23 PCR-positive environmental samples.

Household	Village	# children in household age 5 and under	Source of Positive Sample
1	Majawanga	2	Household furniture
2	Makawa	1	Caretaker’s clothing
3	Njoge	1	Child’s hand
4	Njoge	1	Child’s clothing, Child’s hand
5	Njoge	1	Child’s clothing, Caretaker’s clothing, Bedsheet
6	Njoge	2	Household furniture
7	Njoge	2	Child’s hand, Caretaker’s clothing (x2), Caretaker’s hand, Bedsheet
8	Njoge	2	Caretaker’s clothing
9	Njoge	1	Child’s hand
10	Njoge	2	Child’s clothing, Child’s hand, Bed, Bedsheet
11	Njoge	2	Child’s hand
12	Njoge	2	Child’s hand
13	Njoge	1	Child’s clothing

Obtaining at least one positive environmental swab from the household was associated with village of residence (ranging from 0% in Mkutani and Banyi Banyi to 34% in Njoge, p = 0.011) and presence of ocular infection in the index child (percent positive swabs 34% vs. 3%, p = 0.001) (Table 4). Although not statistically significant, a positive environmental swab was also associated with having an infant <1 year of age in the house, households where secretions were wiped onto household furniture, those in which clothes had been washed ≥3 days ago versus <3 days ago, and those in which bed covers had been washed >1 week ago versus <1 week ago. There was no evidence of an association with trachoma severity, number of children <5 years old in the household, or number of children who slept together.

**Table 4:** Unadjusted associations between positive environmental swabs and household/index child characteristics.

Characteristic	N (households)	% with positive environmental swabs	P value
Village			
Makawa	10	10.0	0.011
Banyi Banyi	10	0	
Majawanga	16	6.3	
Njoge	32	34.4	
Mkutani	12	0	
Severe trachoma:	N (children)		
yes	26	15.4	1.00
no	54	16.7	
Ocular Ct infection			
yes	32	34.4	0.001
no	38	2.6	
More than 1 child <5 years in HH			
yes	41	17.1	1.00
no	39	15.4	
Presence of an infant age <1 year			
yes	19	31.6	0.07
no	61	11.5	
Children <5 years sleep together			
yes	31	22.6	0.23
no	49	12.2	
Caretakers hand used to clean secretions, hands not washed			
yes	67	17.9	0.07
no	13	7.7	
Caretakers clothes used to clean secretions			
yes	66	16.7	1.00
no	14	14.3	
Child’s clothes used to clean secretions			
yes	65	16.9	1.00
no	15	13.3	
Secretions on household furniture			
yes	18	33.3	0.06
no	62	11.3	
Clothes last washed <3 days ago			
yes	41	6.1	0.06
no	39	23.4	
Covers last washed <1 week ago			
yes	60	12.1	0.17
no	20	27.3	

When we included presence of ocular infection in the index child in the model (Table 5, model A), only that variable was statistically significantly associated with obtaining at least one positive environmental swab from a household (OR 22.0; 95% CI 2.31–209). A second model was fit without this variable in order to determine other possible mediators that have an association with a positive environmental swab (Table 5, model B). In this model, presence of an infant <1 year in the household was associated with increased odds of obtaining a positive environmental swab (OR 4.56; 95% CI 1.19–17.5) and washing clothes <3 days prior to the study visit was protective against obtaining a positive environmental swab (OR 0.17; 95% CI 0.03–0.88).

**Table 5:** Adjusted associations between positive environment swabs and household characteristics and reported behaviors.

**Model A.**			
**Characteristic**	**OR**	**95% CI**	**p-value**
ocular infection present in index child	22.0	2.31, 209.0	0.007
Infant <1yo in household	5.31	0.87, 32.5	0.071
Clothes washed <3 days ago	0.14	0.02, 1.03	0.054
**Model B.**			
**Characteristic**	**OR**		**p-value**
Infant <1yo in household	4.56	1.19, 17.5	0.027
Clothes washed <3 days ago	0.17	0.03, 0.88	0.035

## Discussion

Our study found that *C*. *trachomatis* DNA is present in the household environment of children with active trachoma, especially those that currently had ocular infections. These positive environmental samples were found both on household objects and on the hands and clothing of children and their caretakers. The findings do not mean that the environmental source contained live organisms capable of transmission, as we did not test for replicating organisms or load. However, it does suggest the presence at one point of a possible environmental source of infection, and these may have the potential to contribute to disease transmission.

The majority of our positive environment swabs came from the one village, Njoge, with the highest prevalence of trachoma. In all villages, households were only visited if the child had active trachoma and therefore, while we expected to visit fewer households in lower prevalence villages, we expected all households with a child with trachoma to have an equal proportion of positive environmental specimens. However, this was not the case, and in two villages we had no positive environmental specimens in the household. In fact, these two villages had the lowest rates of infection in the index children. The other villages had fewer environmental positive swabs compared to Njoge, although the rates of infection in their index children were similar to the children in Njoge. This difference suggests that the practices of children and caretakers in Njoge may contribute to diverse environmental presence of *Chlamydia*, which in turn may be contributing to the high levels of trachoma. In studies of the contribution of flies to transmission of trachoma and infection, flies seem to play a larger role in transmission in areas of low prevalence, like The Gambia, but not in areas of high prevalence [14, 18]. This difference has been attributed to the multiple routes of transmission in hyperendemic areas such that elimination of any one source has minimal effect on transmission [14]. Our study provides evidence for the potential diversity of environmental sources in hyperendemic areas that may be contributing to transmission.

Not surprisingly, positive environmental swabs were associated with the presence of ocular infection in the index child, which no doubt increased the likelihood that we would find contaminated surfaces. Since the sign of TF can persist long after infection clears, it is possible that degradation of Chlamydial DNA in the environment precluded finding even more positive environmental samples. It is also possible that we missed a fomite in the household if it did not turn up in our questionnaire.

The study of potential sources of environmental contamination of urine samples for diagnosing sexually transmitted chlamydia provides further insight into our findings. Andersson et al swabbed a variety of environmental locations in STI clinic bathrooms, including door handles, locks, doors, walls, toilet flush buttons, and wash basins and taps for evidence of contamination using Aptima 2 Combo Assay (Gen Probe) [19]. They found anywhere from 3.6 to 9.4% of swabs were positive for *C*. *trachomatis*, and included sites like door handles and locks, washbasin taps and wash basin edges. That study was able to show that such sources were also capable of contaminating clean urine samples, as proof of principle. A similar study of clinic surfaces suggested the load of infection was low, but the time of infection was unknown and the load could have declined with time [20]. In studies in the STI clinics, the loads found are small and suggest that transmission of genital chlamydia from these surfaces is unlikely [21]. Nevertheless, the presence of chlamydial DNA points to a non-zero probability, and for trachoma further investigation may be warranted.

We were less likely to find a positive environmental swab in households with increased clothes washing practices. Current hygiene education focuses on face washing to decrease transmission of infected ocular and nasal secretions. Our study suggests that ocular and nasal secretions were wiped using clothing, suggesting that washing clothes and bedding may also be important.

We recognize that this study is limited in its ability to assess associations because of the small number of positive environmental samples obtained. This small number is likely a result of several factors: 1) The low prevalence of trachoma in some of the studied villages, and with fewer index children, we visited fewer households, decreasing the likelihood of finding positive samples; 2) use of active trachoma as a surrogate for infection at the time we scheduled a visit to the house. Ideally, we would have had a rapid test of infection to decide which households to visit, but there was not one available at the time of the study. We have shown the correlation of trachoma and infection even in low prevalence settings which justified the use of this purposeful sampling approach [22]; 3) imprecise sampling area of the object being sampled. We tried to sample the area indicated by the caretaker, but this could be imprecise and subject to recall bias. 4) some of the sampling was based on observation as well as directed by the questionnaire. It may be likely that the observation of a child wiping a hand on a piece of furniture for example, would yield a sample that was more likely positive for its value in being fresh. We cannot differentiate the sampling directed by questionnaire from that by observation, but in any case the fact is we found positive environmental samples from a variety of sources that included ones not easily observed; 4) possible inhibition of samples collected from the environment. The lab carried out DNA extraction on the Roche MagNA Pure LC extraction robot with 200 μl of sample, and in previous experiments using the robot extraction protocol, there were no inhibited samples. We acknowledge that these were all in ocular samples, and that we did not test for inhibition on the environmental samples.

A second limitation is the absence of diversity in a few of the responses; for example, almost all children who were carried, were carried on the caretakers hip. Thus, any association of an environmental positive sample due to that practice is diluted by the commonality of the practice.

Our study provides suggestive evidence that in high prevalence villages, *C*. *trachomatis* may be contaminating environmental objects, including hands and clothing. These findings suggest that behavior change communication around hygiene may need to be enlarged to focus on keeping ocular and nasal secretions off faces and hands, and promoting washing of clothing as well.

## Supporting information

S1 checklist Strobe checklist for cross sectional survey. (DOCX)
